# Supplementary figures and images for: Searching through functional space reveals distributed visual, auditory, and semantic coding in the human brain
Source: PLoS Comput Biol. 2020 Dec 3;16(12):e1008457. doi: 10.1371/journal.pcbi.1008457 (PMC7738169; doi:10.1371/journal.pcbi.1008457)

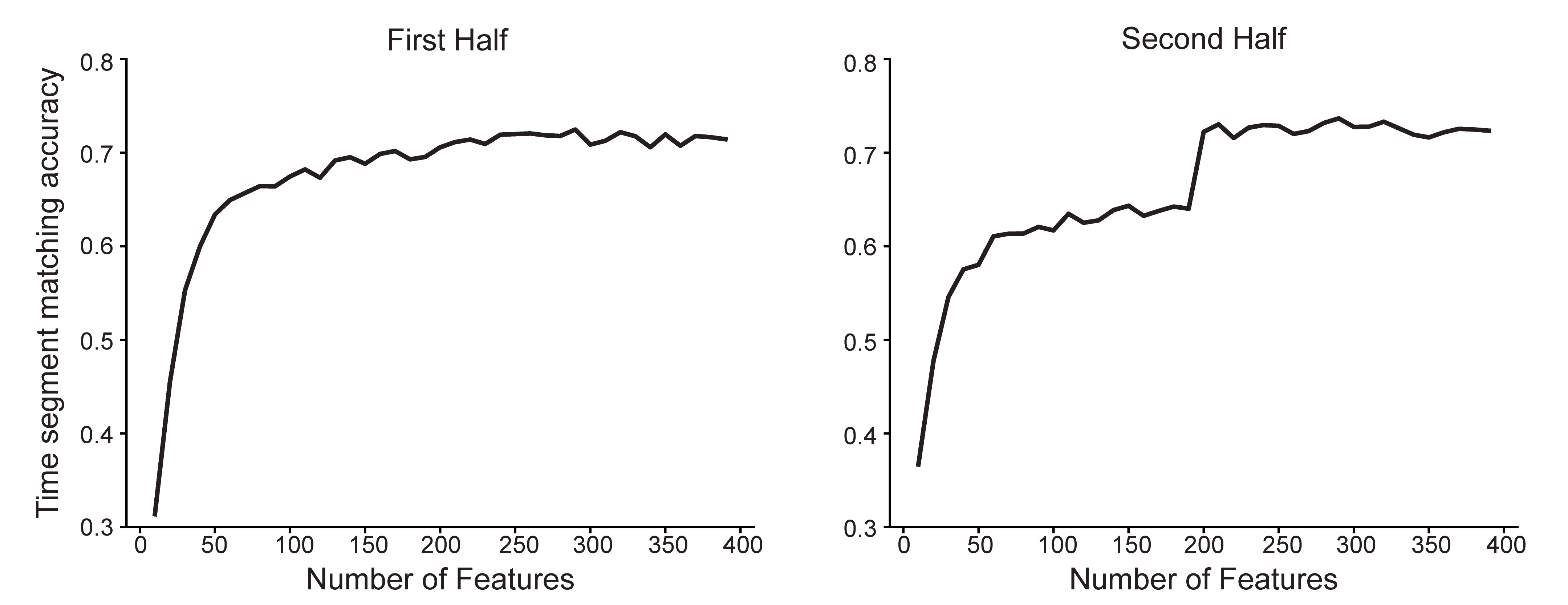

Supplement: S1 Fig — We performed a time-segment matching analysis [8] on the Sherlock data to determine how many SRM dimensions to use. Within the training half of the movie, we trained an SRM with the corresponding number of dimensions (x axis) on one half of the TRs and tested on the second half of the TRs. The goal was to predict from which time window in the movie the test data were obtained (chance = 0.0212). Time-segment matching proportion correct (y axis) plateaued at approximately 200 dimensions considering both folds of the overall analysis. (TIF) [file pcbi.1008457.s001.tif]

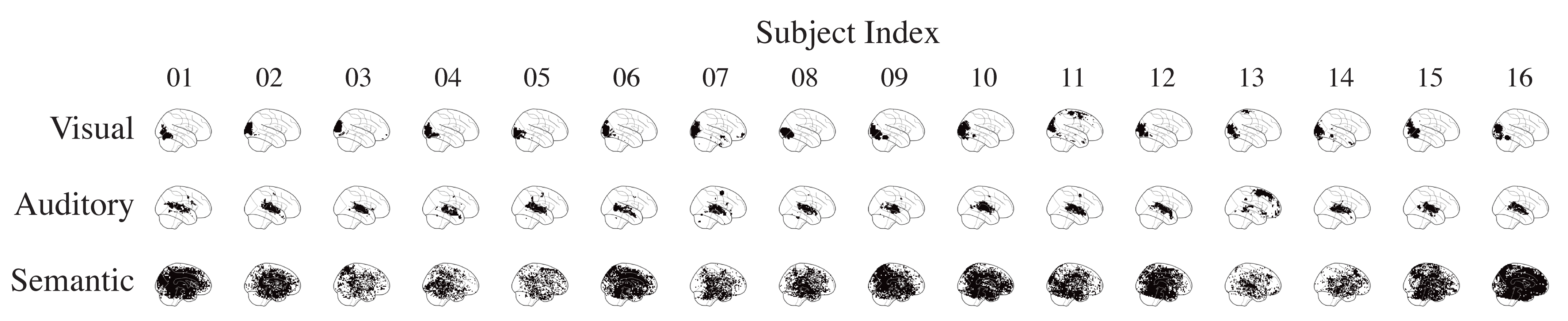

Supplement: S2 Fig — The spatial distribution of the top 1% functional searchlight voxels for each subject and analysis. (TIF) [file pcbi.1008457.s002.tif]

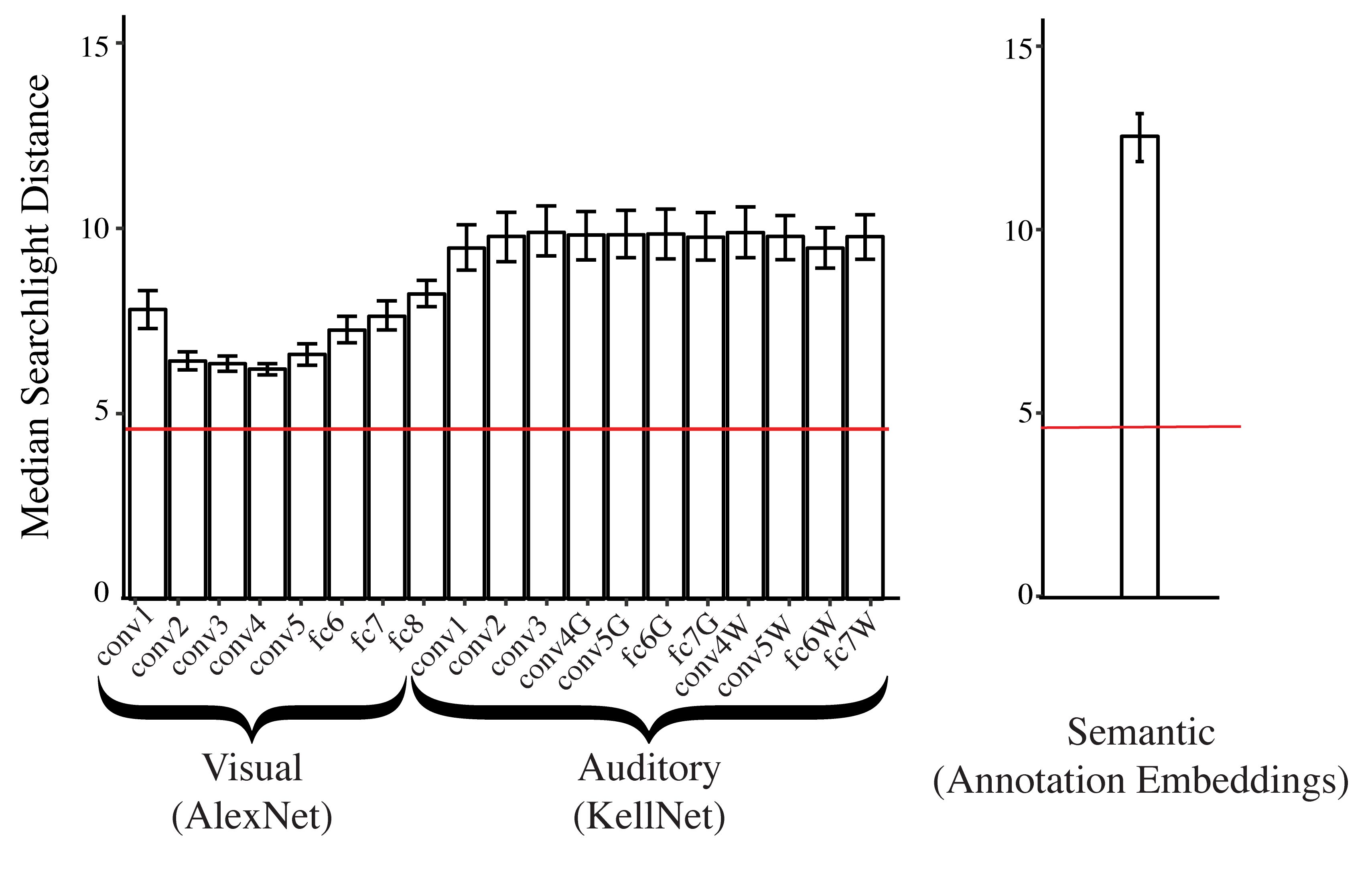

Supplement: S3 Fig — The median Euclidean distance (in voxel units) of the anatomical coordinates of voxels from within the top 1% of functional searchlights in the movie content experiment. Error bars denote 95% CIs across subjects and the red line denotes the median Euclidean distance of voxels from within anatomical searchlights (constant determined by searchlight radius). An appropriate ceiling for these values is the median Euclidean distance in a searchlight consisting of randomly chosen brain voxels (approximately 28 voxels). (TIF) [file pcbi.1008457.s003.tif]

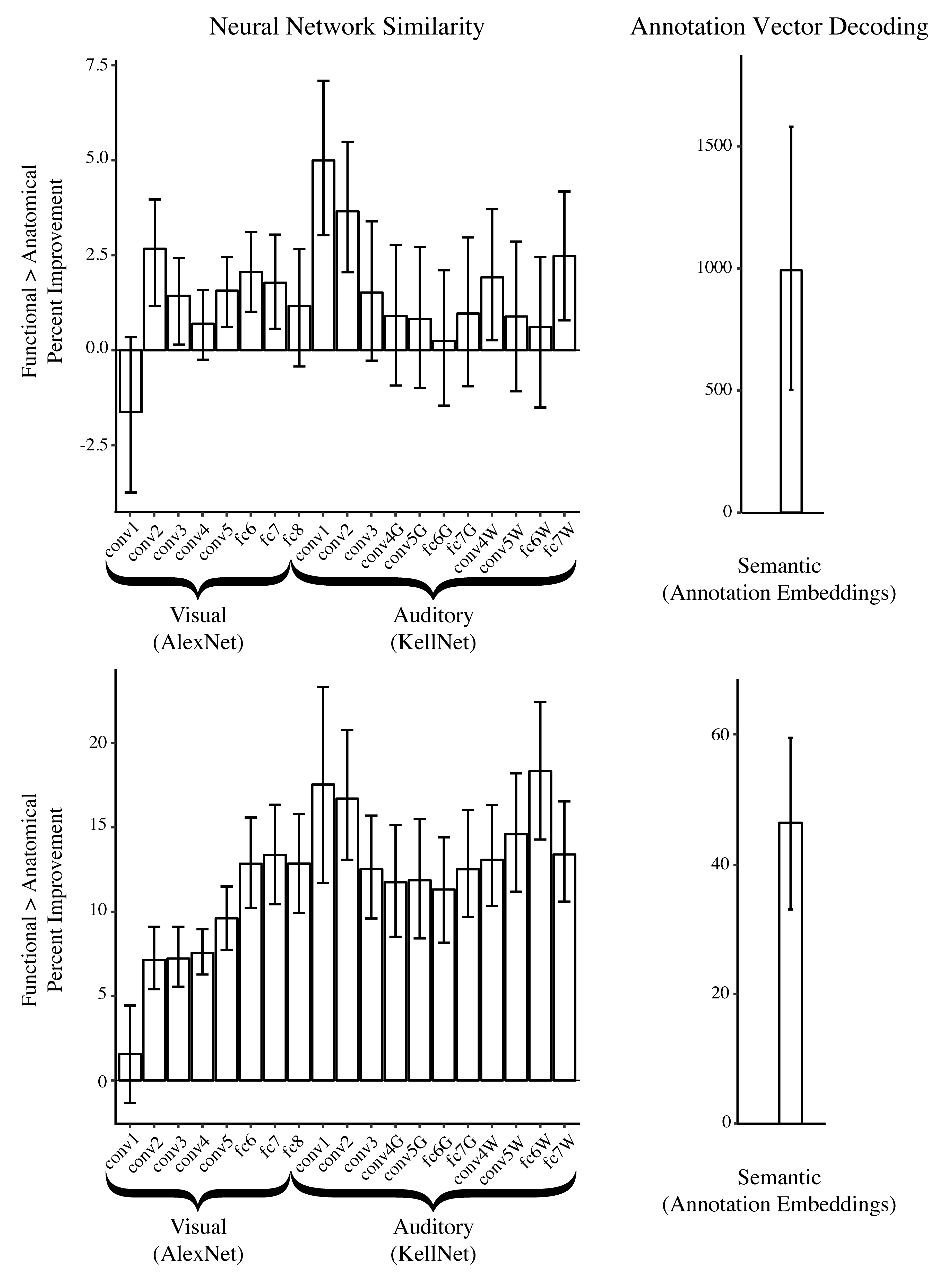

Supplement: S4 Fig — For our main analyses, we chose a searchlight radius = 3 (number of voxels for each searchlight 7 x 7 x 7 = 343). Here we report movie content analyses results for radius = 2 (top row; number of voxels: 5 x 5 x 5 = 125) and radius = 4 (bottom row; number of voxels: 9 x 9 x 9 = 729). A larger radius led to a greater advantage for functional over anatomical searchlights in neural network analyses and a smaller advantage for the NLP analyses. The y-tick values in the top plot for the semantic analysis are a different range because the anatomical searchlight on the natural language embeddings does very poorly when the searchlight radius is low. This speaks to our finding that these natural language representations are widely distributed rather than localized in circumscribed regions of the brain. (TIF) [file pcbi.1008457.s004.tif]

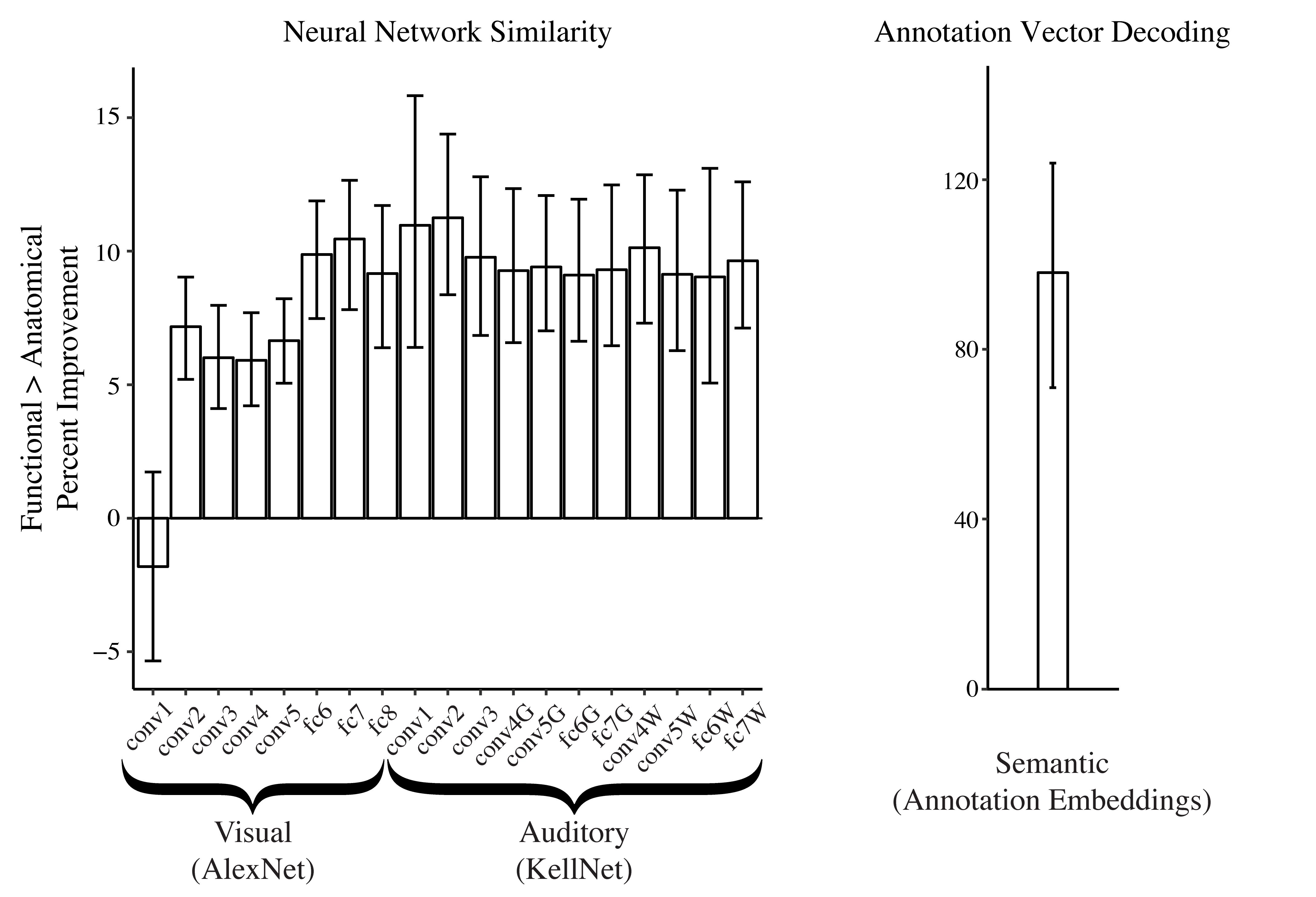

Supplement: S5 Fig — To test whether the improved performance of functional searchlights reflects their ability to aggregate information across hemispheres from bilateral regions that are anatomically distant but functionally homologous (e.g., left and right auditory cortex), we re-ran all of our movie content analyses within one hemisphere of the brain at a time (including fitting SRM) by completely ignoring the other hemisphere. The error bars show 95% bootstrapped CIs. Even in the absence of bilateral information, functional searchlight still consistently outperforms anatomical searchlight. (TIF) [file pcbi.1008457.s005.tif]

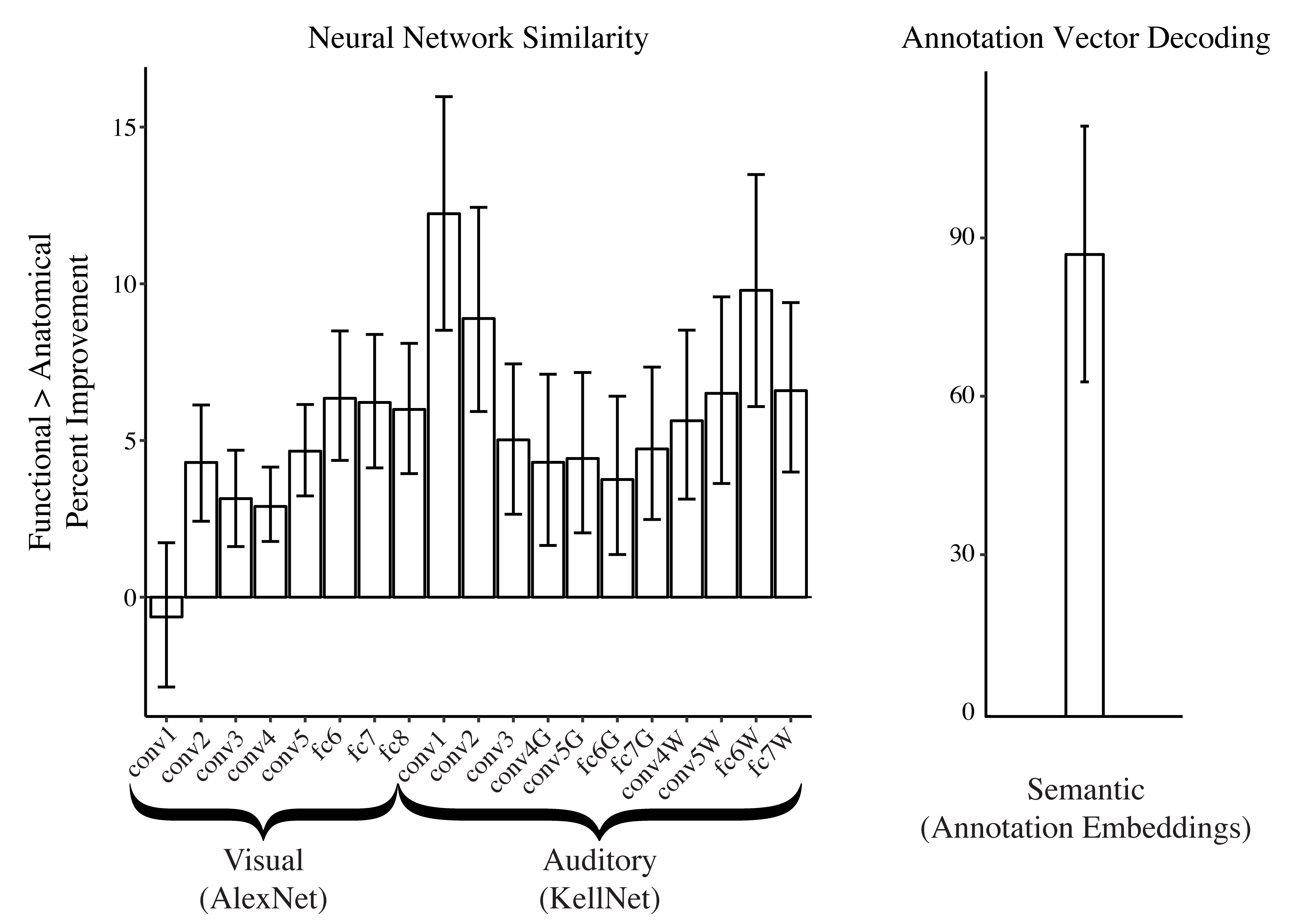

Supplement: S6 Fig — Some anatomical searchlight volumes on the edge of the brain would contain non-brain voxels that need to be excluded, resulting in fewer brain voxels defining the activity pattern. Since we are taking the nearest neighbors in functional space, the number of voxels per functional searchlight is always the same. To ensure functional searchlight is not benefitting from having more voxels in certain searchlights, we ran a version of our movie content analyses where each functional searchlight was forced to contain the exact same number of voxels as the anatomical searchlight for that voxel. The error bars show 95% bootstrapped CIs. Even when we force the number of voxels per searchlight to match, functional searchlight still performs better. (TIF) [file pcbi.1008457.s006.tif]

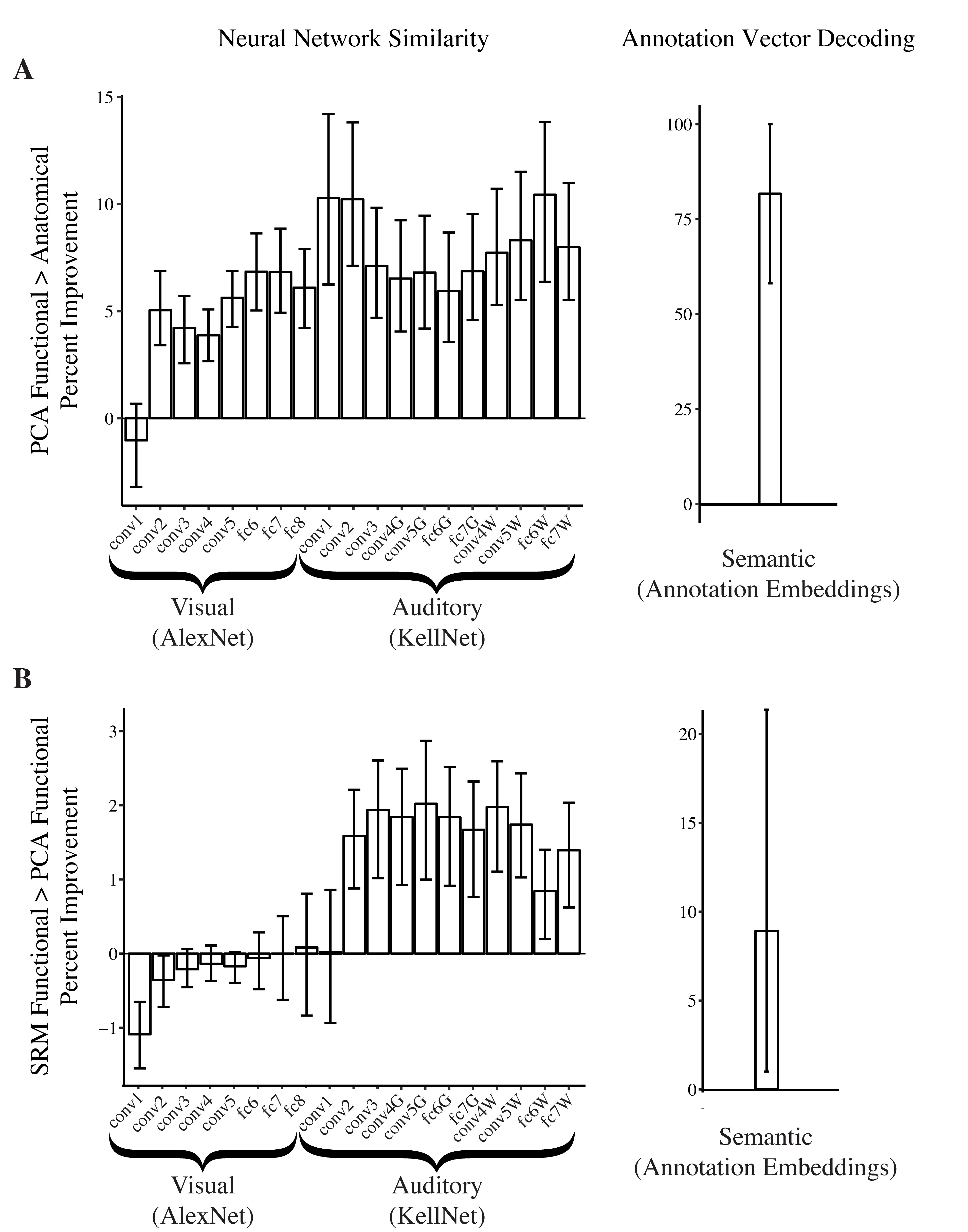

Supplement: S7 Fig — We repeated our analyses on the movie content experiment but replaced SRM with PCA. In particular, we trained a PCA within each subject using the half of the movie that was used for SRM training. Equating the number of dimensions used for SRM, we took the top 200 components (ordered by explained variance) and used each voxel’s loading onto the components as coordinates for functional space. We compared PCA functional searchlight vs. anatomical searchlight (A) as well as SRM functional searchlight vs. PCA functional searchlight (B). PCA functional searchlight outperformed anatomical searchlight. Defining functional space with SRM and PCA yielded similar results for the visual network similarity analyses, but SRM outperformed PCA on both auditory network similarity and the semantic analyses. (TIF) [file pcbi.1008457.s007.tif]

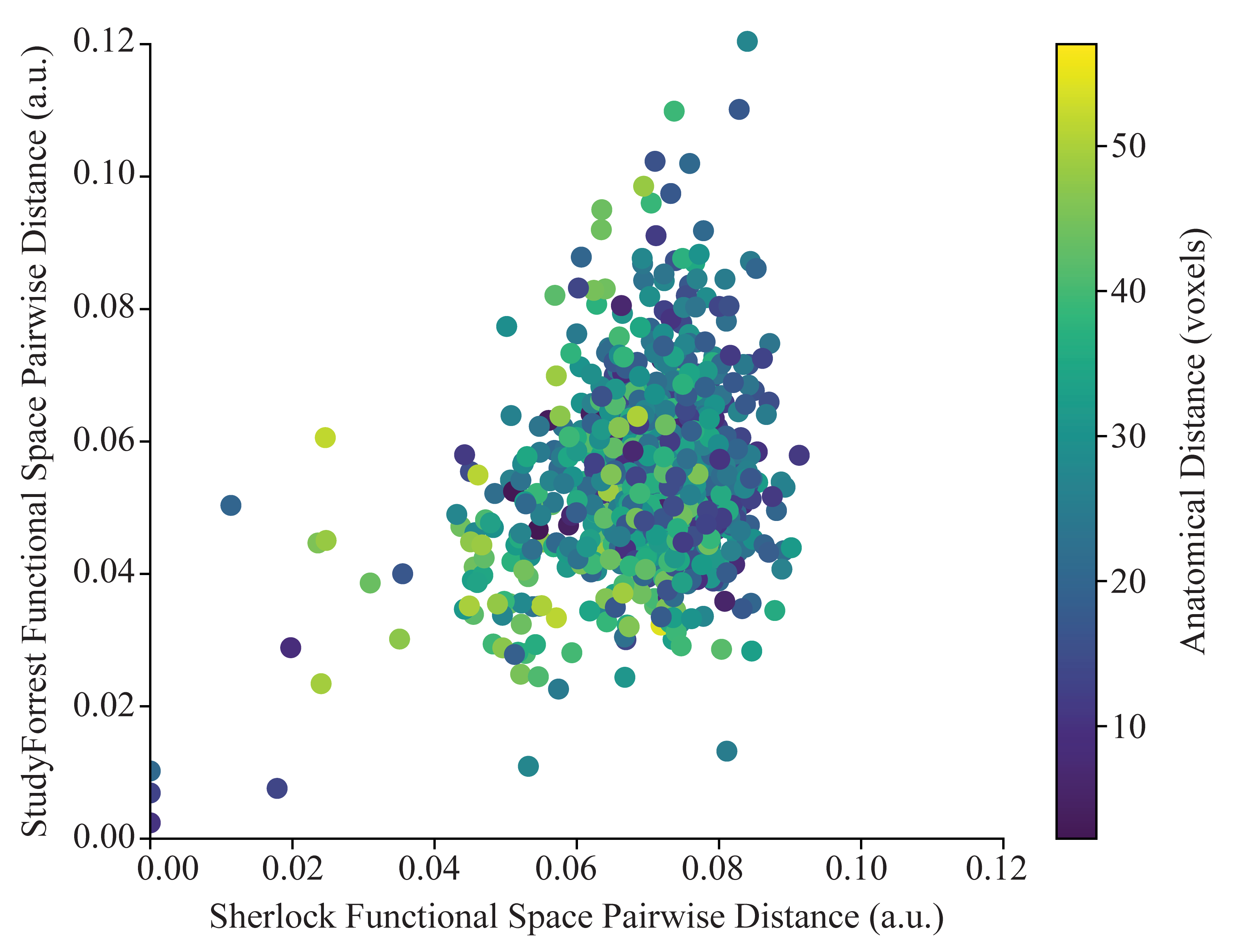

Supplement: S8 Fig — To determine the similarity of the functional spaces constructed by SRM for the Sherlock and StudyForrest datasets, we extracted the Euclidean distances between all voxel pairs in functional space and then correlated these distances between datasets. The overall correlation was estimated by randomly sampling the same 10,000 voxel pairs from the two datasets, calculating the correlation of distances in the sample, repeating 1,000 times with new random samples, and then averaging across iterations. There was a positive correlation overall (r = 0.198). To test statistical significance, we performed a non-parametric randomization test by repeating this process 1,000 times while randomly permuting the voxel distances of the Sherlock dataset each time to populate a null distribution. The true correlation of the datasets was highly significant (p < 0.001). One of the samples used to estimate the overall correlation is visualized in the plot, with each dot depicting one voxel pair and the axes corresponding to their Euclidean distance in the two functional spaces. Color coding by anatomical distance of each voxel pair indicates how dramatically SRM reshaped the brain based on function (i.e., voxels that are anatomically distant can be functionally close, and vice versa). a.u. indicates arbitrary units. (TIF) [file pcbi.1008457.s008.tif]
