# Supplementary material for: Searching through functional space reveals distributed visual, auditory, and semantic coding in the human brain
Source: PLoS Comput Biol. 2020 Dec 3;16(12):e1008457. doi: 10.1371/journal.pcbi.1008457 (PMC7738169; doi:10.1371/journal.pcbi.1008457)
Supplement: S1 Table — These statistics were calculated based on the percent difference of the mean performance of the top 1% functional searchlights over the mean performance of the top 1% anatomical searchlights, using empirically derived chance performance as a baseline. (DOCX) [file pcbi.1008457.s009.docx]

| Analysis name | Mean | Standard deviation | 95% CI lower bound | 95% CI upper bound | p-value |
| --- | --- | --- | --- | --- | --- |
|  |  |  |  |  |  |
| Visual RSA-conv1 | -0.198 | 4.731 | -2.542 | 2.102 | 0.5646 |
| Visual RSA-conv2 | 5.909 | 3.542 | 4.179 | 7.611 | <0.0001 |
| Visual RSA-conv3 | 4.597 | 3.111 | 3.127 | 6.183 | <0.0001 |
| Visual RSA-conv4 | 4.160 | 2.424 | 3.032 | 5.366 | <0.0001 |
| Visual RSA-conv5 | 5.834 | 2.831 | 4.519 | 7.248 | <0.0001 |
| Visual RSA-fc6 | 8.071 | 3.859 | 6.247 | 10.023 | <0.0001 |
| Visual RSA-fc7 | 7.943 | 4.251 | 5.967 | 10.085 | <0.0001 |
| Visual RSA-fc8 | 7.149 | 4.206 | 5.145 | 9.176 | <0.0001 |
|  |  |  |  |  |  |
| Auditory RSA-conv1 | 12.538 | 8.424 | 8.334 | 16.622 | <0.0001 |
| Auditory RSA-conv2 | 10.436 | 6.644 | 7.384 | 13.854 | <0.0001 |
| Auditory RSA-conv3 | 7.123 | 5.366 | 4.651 | 9.904 | <0.0001 |
| Auditory RSA-conv4G | 6.458 | 5.442 | 3.946 | 9.256 | <0.0001 |
| Auditory RSA-conv5G | 6.861 | 5.592 | 4.240 | 9.683 | <0.0001 |
| Auditory RSA-fc6G | 5.895 | 5.111 | 3.518 | 8.521 | <0.0001 |
| Auditory RSA-fc7G | 6.773 | 5.237 | 4.325 | 9.445 | <0.0001 |
| Auditory RSA-conv4W | 7.678 | 5.381 | 5.241 | 10.536 | <0.0001 |
| Auditory RSA-conv5W | 8.210 | 6.172 | 5.392 | 11.346 | <0.0001 |
| Auditory RSA-fc6W | 10.783 | 7.914 | 6.878 | 14.588 | <0.0001 |
| Auditory RSA-fc7W | 8.244 | 5.577 | 5.740 | 11.146 | <0.0001 |
|  |  |  |  |  |  |
| Semantic decoding | 94.155 | 45.165 | 70.819 | 116.024 | <0.0001 |
|  |  |  |  |  |  |
| Image classification | 12.576 | 6.151 | 9.615 | 15.917 | <0.0001 |
|  |  |  |  |  |  |
