# Supplementary material for: Searching through functional space reveals distributed visual, auditory, and semantic coding in the human brain
Source: PLoS Comput Biol. 2020 Dec 3;16(12):e1008457. doi: 10.1371/journal.pcbi.1008457 (PMC7738169; doi:10.1371/journal.pcbi.1008457)
Supplement: Attachment — Submitted filename: Kumar_PCB_Revision_Letter_Final3.pdf [file pcbi.1008457.s010.pdf]

Dear Drs. Jbabdi and Marinazzo,

Thank you for organizing the review of our manuscript “Searching through functional space reveals distributed visual, auditory, and semantic coding in the human brain” and for inviting us to submit a revision.

Below we provide point-by-point responses to all of the reviewer comments and highlight where in the manuscript changes were made. We are grateful for the highly constructive suggestions from the reviewers, which we believe considerably improved the manuscript. We hope that you agree and that our manuscript might now be ready for publication.

Sincerely,

Nick Turk-Browne (on behalf of all authors)

--

Reviewer #1: Authors in this paper propose an alternative to traditional searchlight based search for information called functional searchlight. Gist of it is to use a set of voxels that have high weight in some latent shared dimensions that explain most variance during a movie viewing task. They show that these sets of voxels as opposed to anatomically selected sets have higher correlation with representations of different layers of DNNs for audio and visual input and higher accuracy in decoding movie scenes.

> We thank the reviewer for their extremely helpful comments that prompted large-scale improvements to the paper.

Manuscript is clearly written and well structured and easy to follow.

I have major concerns about the proposed method, its usability in many scenarios compared to traditional searchlight, and the claim of distributed representation being confounded by the stimulus used.

Proposed method:

- Authors present this as a novel idea/method but this has been presented and used by the original papers on Hyperalignment: Haxby et al (2011) restricted to VT and then expanded to whole brain in Guntupalli et al (2016)

> The work of Guntupalli et al. (2016) and Haxby et al. (2011) is relevant and similar to our work, because we all use functional alignment to improve MVPA. However, it is critical to note that in these two papers hyperalignment was performed within regions of interest/searchlights, whereas in our work, functional alignment was performed prior to searchlight analysis. This is an important difference that uniquely allows our method to identify distributed representations in the brain. For instance, if a representation for some part of the movie is distributed across large swaths of the brain, then our method may group those anatomically disparate voxels together, allowing them to be part of the same searchlight. In contrast, the methods described in Guntupalli et al. (2016) and Haxby et al. (2011) would be unable to detect such distributed information. What is common across these methods is that if the voxels carrying information about a representation are not the same across participants then both methods could still extract the information. Our method is unique in that the power of searchlight analysis can be utilized while also integrating representations spanning the whole brain. Moreover, although performing functional alignment within a region of interest/searchlight allows for small differences in localization of function, our method allows for gross changes in anatomical alignment — what might be expected, for example, from lateralized functions

between left- and right-handed individuals or developmental reorganization across children of different ages. Hence, we believe that the nature of our method is different that these important works. Nonetheless, we agree with the reviewer that we did not do enough to explain our method in the context of the literature, including these two studies.

Manuscript change:

> "This method has similarities to approaches used previously but is unique in its potential to discover distributed representations. Functional alignment has been performed within a region of interest [9] and within searchlights [10] to create denoised representations for input to MVPA. In this case, functional alignment is a preprocessing step to remove sources of noise not shared across participants and to compensate for small differences in anatomical alignment of voxels within the region of interest/searchlight. However, these methods are different from ours: we perform functional alignment on the whole brain (rather than a subset of voxels) to reorganize all voxels (rather than to denoise them) prior to running searchlight MVPA in this reorganized space. Hence, our method is uniquely positioned to reveal broadly distributed neural representations." p. 6

- Using top-weighting voxels in the reduced shared space and treating them as unit of information representation is problematic for two reasons: 1. these could be driven by correlations in the movie, which are by design correlated across domains, 2. without showing that these are truly distributed representations, one needs to show the evidence in an experiment devoid of such correlated stimulus. For example, in the above mentioned papers, they do so on controlled independent experiments with still images, functional localizers, and retinotopy. Extraordinary claims like this require extraordinary evidence.

We would like to clarify that we are not using top-weighted voxels in the shared space, but organizing which voxels are in a searchlight based on whether they have *similar* weights. A searchlight is run centered on every voxel in both anatomical and functional spaces using the same voxel time course information. In functional searchlight, which voxels contribute to a searchlight is determined by the similarity of the weights used to transform voxels into shared space. In other words, all voxels end up somewhere in shared space and contribute to multiple searchlights, even when they have low weights.

We realized that the confusion might arise because we selected the top 1% of searchlights to evaluate performance. The top 1% doesn't refer to the weights of the voxels in the shared space. In fact, these top performing searchlights tend not to have voxels with the highest weights. Rather, the top 1% is based on the performance of these voxels in the similarity and decoding analyses. In other words, by taking the top 1% we are asking how good are the best voxels from the whole-brain anatomical searchlight and comparing them to an equivalent number of best voxels in the functional searchlight. Nonetheless, we agree that this important nuance should be clearer.

Manuscript changes:

> "With the exception of voxels that have exactly zero weight to the shared space, *all* voxels are used in the functional searchlight, not just voxels with high shared space weights." p. 15-16

> "It is also important to note that functional searchlight is doing this exhaustive search on exactly the same brain voxels and time series as anatomical searchlight, except the voxels that comprise each searchlight are different." p. 5

> "When we take the top 1% of each, there will be an equivalent number of searchlights in each method used for evaluation." p. 8

Beyond these clarifications, the reviewer raises an important point about correlations in the movie. As we understand it, the concern is that grouping voxels by their weights onto shared space will be impacted by these correlations and that testing with movie data containing the same kinds of correlations could bias performance. Testing on completely held-out movie data ameliorates these concerns, at least insofar as different parts of the movie have different correlations or noise. However, in support of the reviewer's concern, the training and test movie segments contained similar content that may have resulted in similar signal correlations.

As suggested by the reviewer, in order to ensure that our results are not driven by correlations in the movie, we added a second dataset to the revision (StudyForrest). This dataset consists of fMRI from subjects watching a naturalistic audiovisual stimulus (*Forrest Gump*) as well as a localizer experiment in which subjects are shown static images from six different categories in a block design: bodies, faces, houses, small objects, landscapes, and scrambled images. This fits the reviewer's criteria of a controlled experiment with still images independent from movie data. We fit a functional space on a subset of the movie data (equivalent in size to the data analyses from the Sherlock dataset) via SRM and tested our method on decoding image category from brain activity on the subsequent localizer experiment. We compare decoding performance to traditional anatomical searchlight with no SRM performed. The results have been incorporated in Figure 2c-d and are described in the main text.

To summarize, every subject had better classification of object categories in the top 1% of functional searchlights compared with the top 1% of anatomical searchlights. The voxels in these top searchlights were again more distributed in functional searchlight than anatomical searchlight. By replicating our central findings, this analysis shows that our method works on an independent controlled experiment. We are very grateful to the reviewer for this suggestion, as the inclusion of a second dataset adds substantially to the paper.

Updated Figure 2:

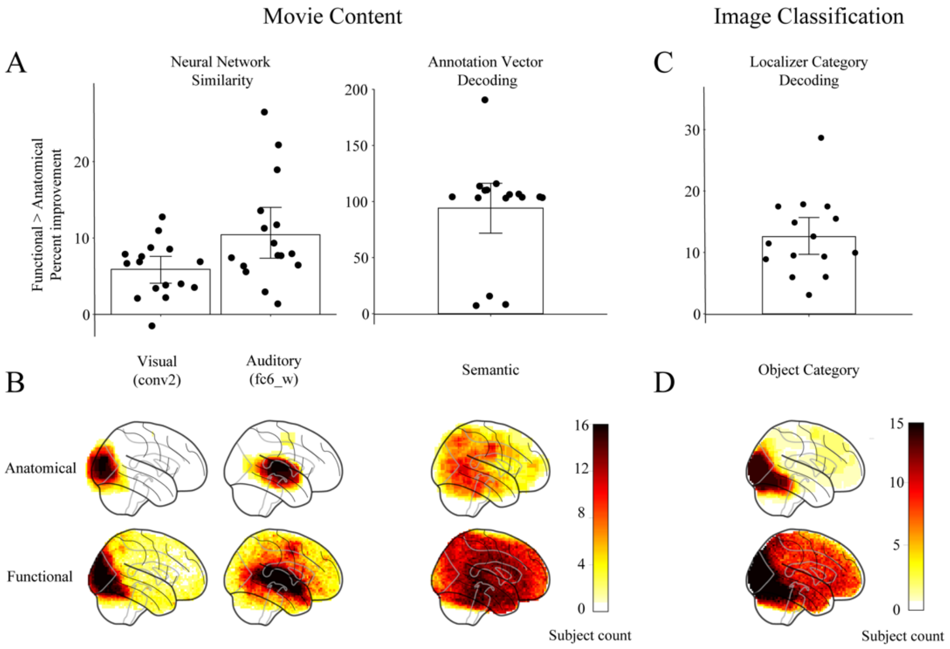

Fig 2. Enhanced performance of multivariate analysis with functional searchlight.
In each subject, we calculated the percent improvement of functional searchlight over anatomical searchlight for the top-performing 1% of searchlights from each approach. (A) Each dot represents a subject's percent improvement for example layers in the AlexNet visual network (conv2) and KellNet auditory network (fc7_W), as well as for annotation vector decoding. Error bars depict 95% CIs from bootstrapping. Absolute performance levels for each searchlight type and non-parametric baselines can be found in Fig S3. (B) For the sensory analyses, we visualize which voxels contained model-based information by depicting the count of the number of subjects for whom that voxel contributed to one or more of the top 1% of their functional and anatomical searchlights. For the semantic analysis, we do the same but only visualize the center voxels of the top 1% of searchlights to avoid clutter. (C) We compared the two methods on a localizer experiment by attempting to classify brain activity evoked by images from six categories: bodies, faces, houses, objects, landscapes, scrambled. Each dot represents percent improvement from chance of the mean top 1% searchlight accuracy. Error bar depicts 95% CI from bootstrapping. (D) We visualized locations of all voxels that contribute to the top-performing searchlights for category decoding.

Additional manuscript changes:

> "To ensure that the utility of functional searchlight is not restricted to naturalistic movies, we analyzed the StudyForrest dataset [18], in which subjects watched the Forrest Gump movie and completed a block-design category localizer paradigm. We ran functional and anatomical searchlight analyses to decode localizer image category from voxel activity patterns (six categories: bodies, faces, houses, small objects, landscapes, scrambled images) and found that the functional searchlight reliably outperformed anatomical searchlight (Fig 2C). Even for this more standard form of image classification, which is often assumed to capture relatively localized representations in category-selective regions, the voxels that contributed to the top 1% of searchlights were much more distributed in functional searchlight than anatomical searchlight. (Fig 2D)." p. 10-11

> Added "StudyForrest dataset" section to Methods, with details about the participants, materials, and analyses.

“This approach revealed a tighter correspondence between the human brain and computational models as well as better results on a more standard image category decoding paradigm.” p. 12

- As authors acknowledge in their introduction, people have done whole brain decoding before and as they rightly pointed out, due to curse of dimensionality most methods rely on dimensionality reduction methods such as PCA/ICA etc. Here, they used SRM, a variant of hyperalignment, to reduce dimensionality, but do not explain why they do that instead of, say, using PCA of every individual's data and showing that high-weighting voxels for top-N PCs are distributed (which they will be). I understand the reasons due to my expertise but it is not clear in the paper.

We thank the reviewer for the opportunity to clarify. SRM is merely a kernel inside our larger framework of doing searchlight analysis on a space where we have rearranged voxel locations based on loadings onto latent variables. We chose SRM because it is a powerful tool for hyperalignment, outperforming other hyperalignment methods (Chen et al., 2017), particularly because it finds a low-dimensional representation that is shared across participants. That said, there are other methods that provide weightings of voxels onto lower-dimensional latent variables besides SRM, such as PCA (which is a parent method of SRM but does not take account of shared between-participant variance). To address this point, we re-ran our movie analyses with PCA instead of SRM.

New Figure S9:

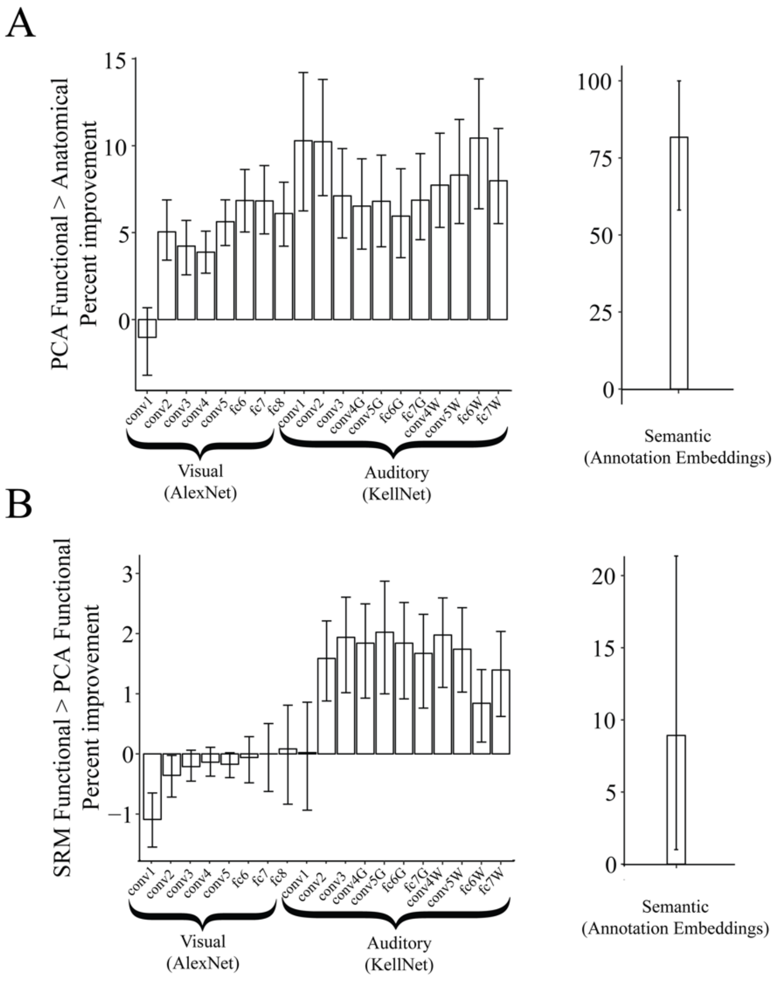

Fig S9. Substituting PCA when defining functional space.
We repeated our analyses on the movie content experiment but replaced SRM with PCA. In particular, we trained a PCA within each subject using the half of the movie that was used for SRM training. Equating the number of dimensions used for SRM, we took the top 200 components (ordered by explained variance) and used each voxel's loading onto the components as coordinates for functional space. We compared PCA functional searchlight vs. anatomical searchlight (A) as well as SRM functional searchlight vs. PCA functional searchlight (B). PCA functional searchlight outperformed anatomical searchlight. Defining functional space with SRM and PCA yielded similar results for the visual network similarity analyses, but SRM outperformed PCA on both auditory network similarity and the semantic analyses.

The top plot shows the percent improvement of the functional searchlight (where voxels were rearranged based on PCA loadings) over the anatomical searchlight. PCA functional searchlights outperformed anatomical searchlights. To determine under which conditions SRM is a *better* kernel in the functional searchlight approach, we compared SRM functional searchlight performance with PCA functional searchlight performance. The two were not significantly different for visual network analyses (except for conv1, where PCA was better), but differed significantly for auditory and semantic analyses. In other words, using SRM to extract clean representations of signal, which it does by aggregating across participants, was most important outside of visual cortex. We emphasize in the revision that SRM is not the only option and that our functional searchlight method can flexibly accommodate other kernels.

Manuscript change:

> "Although we used SRM to create the functional space in the main analyses, a lower-dimensionality representation can be learned in other ways, such as through principal components analysis (PCA). We explored replacing SRM with PCA in functional searchlight in Fig S9. As with SRM, PCA functional searchlight outperformed anatomical searchlight. However, SRM performed significantly better than PCA for the auditory and semantic analyses (though not the visual analyses). These results indicate that it is not always important to use a procedure that aggregates across participants (like SRM) in functional searchlight. In fact, because PCA is run within an individual, it can be used for any type of fMRI design (e.g., event-related trials in pseudo-random order across participants) and not just movie data where there is known correspondence in timing and content across participants. Nevertheless, SRM performed better than or equal to PCA almost across the board, suggesting that it is a good default when such correspondence exists." p. 10

Utility of the method:

- Searchlight as originally proposed is useful and popular for good reasons. It is comparable across studies and not conflated by correlations in the stimulus.

We agree with this and highlight that our motivation for this new method was to provide an additional tool with the same uses as the searchlight but with the added benefit of sensitivity to distributed representations.

- I am not sure how the method proposed here can be used by a generic fMRI study. Authors do not give any evidence that these functional SLs are meaningful and comparable across studies.

In our initial version of our paper, we presented evidence that functional searchlights were more similar to CNN models of vision and audition, as well as semantic models,

than anatomical searchlights. In recent years, the similarity of brain activity to model-based predictions from CNN's has become a major focus (Guclu et al 2015; Long et al 2019; Kietzmann et al 2019; Horikawa et al 2017; Yamins et al 2014). Hence, we believe showing a substantial improvement in this similarity is a meaningful contribution. We also show how to revert the functional searchlights back into anatomical space, which allows distributed representations to be visualized and compared across studies.

In the revision, we further demonstrate the usefulness of functional searchlights in two new ways: First, we show that our method works well (and better than the standard anatomical searchlight) on a controlled category localizer experiment independent from the audiovisual movie used to establish the functional space. Second, we show that our method works well with PCA, suggesting that it can be used even when there is no movie stimulus and the stimuli and trial order vary across participants.

Manuscript change:

> "An important methodological conclusion of this work is that conversion to functional space is worthwhile as a preprocessing step prior to searchlight analysis. Our results in Fig S9 indicate that SRM is not absolutely necessary for creating the functional space because a within-subject dimensionality reduction method, such as PCA, can also be effective." p. 12

- If the idea is to use them for decoding or stimulus feature modeling, then it has already been proposed and used elsewhere and there is nothing novel about that.

The reviewer is right in that we are using the functional searchlight as an exploratory method to find a set of voxels for decoding and stimulus feature modeling, just like the standard anatomical searchlight has been used. The novelty of our method is that (a) it can be used to find a distributed set of voxels for this purpose, whereas the standard anatomical searchlight cannot; and (b) functional searchlight outperforms anatomical searchlight in almost every case we considered.

Manuscript change:

> "The novelty of our method is both that it can be used to find a distributed set of voxels for this purpose (whereas standard anatomical searchlight cannot) and that functional searchlight outperforms anatomical searchlight in almost every case we considered." p. 12

- Anatomical/functional ROIs provide natural regularization and therefore generalize beyond the training distribution. Authors provide no evidence that their method generalized beyond training distribution. No, part 2 of same movie doesn't count. They both have similar correlations.

We appreciate this point, which is similar to a concern raised earlier. Indeed, learning a shared response based on one part of the movie will mean that applying that shared response to another part of the same movie will likely be a good fit. This is true of many past studies using functional alignment methods (Chen et al. 2016; Turek et al. 2017). However, we stress that non-independence is not a concern here: the data used for testing in the searchlights are never seen during training. That is, the mapping into functional space uses fixed, time-invariant weights on each voxel and even if they had embedded some notion of the timecourse of events, the functional space is trained without knowledge of the test data so the training data cannot have helped. The fact that reorganizing voxels based on shared function in one part of the movie improves the

performance of searchlight analyses in the other part, relative to anatomical searchlights, is part of the point. This is not so different from performing feature selection during cross-validation, wherein the training set on a given fold is mined in a nested manner for features conducive to better performance on a test partition of the same dataset.

That said, the reviewer's reasonable question is about generalization — the extent to which a functional space can be learned that improves the performance of analyses of more traditional task designs. The addition of the StudyForrest dataset provides evidence that our approach has value in such situations: the functional space from an audiovisual movie stimulus enhances searchlight performance in decoding the category of static images from separate localizer data.

Problems with claims:

- SRM and the original hyperalignment are known to clean up signal related to shared stimulus content, acting like a filter. To give a hypothetical example, imagine using a stimulus with no audio for deriving shared model, it invariably reduces weights to voxels in auditory regions of STS, this will improve SNR for subsequent analyses. So, it is important to compare anatomical against SRM filtered data: Guntupalli et al. (2016) did that by retrojecting the hyperaligned data into back into subjects (see their papers for specifics).

The reviewer has described one use of SRM, but we present here a novel use of SRM. In particular, we show that the *loadings* of voxels onto shared space can be useful in of themselves, without using the data in shared space (i.e., the weight matrices rather than the functionally aligned data). That is, we don't use SRM to filter the data and only use it to rearrange which voxels go into which searchlights — voxel timeseries are untouched. Our functional searchlight method therefore does not benefit from "cleaned up" signal, and instead has access to the same data as the anatomical searchlight at the level of individual voxels, the only difference being that these voxels have new coordinates. Our manuscript change in response to the reviewer's first point articulates the difference between Guntupalli et al. (2016) and our work.

- Another problem is with decoding movie scenes. If we only look at a SL in calcarine sulcus, we will not be able to differentiate two scenes with same visual but if we include voxels in Heschl's gyrus, we can use any discriminatory auditory information to differentiate the two. So, the results about better decoding of movie scenes with voxels distributed across the brain compared to the same number of localized voxels is trivial and shown before in aforementioned papers with the acknowledgement of the reasoning.

We agree with the reviewer in their logic behind differentiating movie scenes and in fact this thinking motivated the study. Although we do not decode movie scenes in the present work (we regress vector embeddings of natural language annotations), we could apply similar logic to our main visual analysis, RSA with AlexNet.

The reviewer is correct that including voxels from non-visual regions may boost discriminability in the representational similarity matrix for time-points corresponding to scenes with highly similar visual features. However, this scenario is mostly hypothetical: For a voxel in auditory cortex to be part of a functional searchlight containing predominantly visual voxels (or vice versa), those voxels would have to load very similarly onto the shared features in the training half, that is, represent the same content. This does not typically occur for movie stimuli and did not occur here. To the extent that "visual" and "auditory" voxels have very similar loadings, it would stand to reason that they represent higher-order multimodal or amodal content (e.g., semantic features) that is distributed across anatomically circumscribed regions. We respectfully disagree that this is trivial.

Overall, there are many issues which the authors fail to acknowledge or present in the paper. Discussion section section should cover these in detail so the readers know the caveats.

We thank the reviewer for their many helpful comments, which although challenging and requiring extensive new analyses, made the manuscript stronger.

Minor comments:

- Results are not reported in text beyond qualifiers. Please provide the numbers and statistical test results in test and actual measures to assess the effect sizes.

We added the means, standard deviations, 95% confidence intervals, and non-parametric bootstrapped p-values of the main results in a new Table S1.

Figure 2: Mentioning actual correlation values for RSA and decoding in caption will give indication effect sizes.

We have also included these values in Figure S5, along with their empirically estimated chance values.

Figure S4 top right panel's Y-tick values seem wrong

Although the y-tick values in that panel are very different, they are accurate. It is just that the anatomical searchlight analysis on the natural language analysis does very poorly when the searchlight radius is low. This speaks to our finding that these natural language representations are widely distributed rather than localized in circumscribed regions of the brain. We will emphasize this point in the caption (note: this figure has now become S6).

Manuscript change:

> Figure S6 caption: "For our main analyses, we chose a searchlight radius = 3 (number of voxels for each searchlight 7 x 7 x 7 = 343). Here we report movie content analyses results for radius = 2 (top row; number of voxels: 5 x 5 x 5 = 125) and radius = 4 (bottom row; number of voxels: 9 x 9 x 9 = 729). A larger radius led to a greater advantage for functional over anatomical searchlights in neural network analyses and a smaller advantage for the NLP analyses. The y-tick values in the top plot for the semantic analysis are a different range because the anatomical searchlight on the natural language embeddings does very poorly when the searchlight radius is low. This speaks to our finding that these natural language representations are widely distributed rather than localized in circumscribed regions of the brain." p. 32

- All the analyses the authors did were done in prior work, please acknowledge that in introduction.

We appreciate the feedback and have added more citations and discussion of prior work. At the same time, we feel that this comment is not very charitable and that there are several novel aspects of our work. The reviewer has encouraged us to describe these aspects more explicitly, for which we are grateful.

Reviewer #2: This paper describes a new method for finding distributed representational structure in functional brain imaging data, and compares it to the widely-used anatomical searchlight approach. The new method first maps BOLD data into a low-dimensional functional space using shared-response mapping, so that voxels showing similar functional profiles are situated near one another in the functional space. They then apply the searchlight technique,

using the functional proximities to define searchlights rather than anatomical proximities. The authors use anatomical and functional searchlights to decode visual, auditory, and semantic structure in the Sherlock dataset, comprising functional brain images from 16 subjects as they watched an episode of the TV show Sherlock. They show that the new method can better predict target similarity matrices in all cases, and suggest a more anatomically distributed signal relative to the standard approach, consistent with a developing view that neuro-cognitive representations are more distributed in the brain than classical view have suggested.

This work contributes to a developing literature innovating new data-science approaches to learn more about how information is encoded in neural systems. As the authors note in the introduction, a long-standing debate in cognitive neuroscience concerns the degree to which different functions should be viewed as anatomically localized or distributed. In brain imaging, textbook findings consistent with localizationist views may be an artifact of widely adopted statistical methods that are only capable of finding localized signal, a point that has been emphasized by a variety of groups in recent years. The approach to finding less localized signal in the current work was new to me, however, and represents a potentially important contribution to this literature. The paper is very clearly and concisely written, and the results seem compelling. I have some suggestions for further clarifying and elaborating the approach and the findings that I hope may be useful in maximizing the paper's impact.

We thank the reviewer for their positive comments and helpful suggestions.

1) The results are reported in terms of percent improvement over the anatomical searchlight method, but it would also be useful to see the empirical model accuracy. Often searchlight methods report results that are statistically distinguishable from a null result, but nevertheless weak (e.g. correlation between neural and target RDM of ~0.05, reliably non-zero but....). It would be useful to see what the actual correlation between predicted and true RDMs are for the top 1% of searchlights here. I understand that the point of the paper is to show that functional searchlight is reliably better-performing than anatomical, but for others adjudicating whether to adopt this or other approaches, the raw fits would be useful to see.

We agree about the importance of reporting the raw performance levels in addition to the percent improvement. These have been included in Figure S5 along with empirically estimated chance values.

2) A central finding is the more broadly-distributed signal revealed by the functional-searchlight result, which also shows greater accuracy. This is especially noteworthy as both the size of the searchlight and the number of "good performing" searchlights are matched between functional and anatomical approaches---so the broader signal does not reflect, for instance, more searchlights being "selected." But, I think there are two importantly different potential explanations. One is that searchlights in all subjects are grouping together anatomically-distal voxels; the other is that the selected voxels vary quite alot in their location across subjects. It would be useful to know how consistent the anatomical distribution of selected voxels is across subjects.

We thank the reviewer for this excellent suggestion. We have now added Figure S3, which contains the individual locations of the selected voxels in the top 1% of functional searchlight for each subject. These 16 maps for each condition are summed in Figure 2B. From these plots, it can be seen that the distributed voxels in Figure 2 are fairly consistent across individuals in the visual and auditory analyses. In the semantic analysis, the voxels are highly distributed within subject, but the specific spatial distribution varies across subjects.

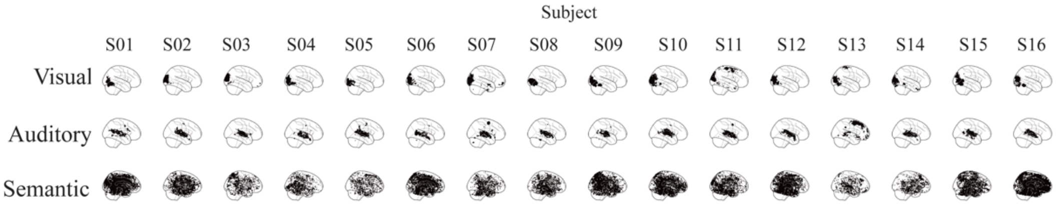

Fig S3. Distributed representations in individual subjects.
The spatial distribution of the top 1% functional searchlight voxels for each subject and analysis.

We also added Figure S4 which compares the median Euclidean distance in anatomical space of the voxels from the top 1% of functional searchlights. If the top-performing functional searchlight voxels came from small local regions, their Euclidean distance should be closer to the (fixed) median distance of voxels within anatomical searchlights. Figure S4 shows that the functional searchlights contain more distributed voxels than anatomical searchlights. This analysis does not directly distinguish between larger contiguous regions and more disparate voxel sets. The analysis of searchlight size (Figure S6), suggests that the benefit of functional searchlight does not derive from capturing larger contiguous regions, as giving anatomical searchlights a greater radius, and thus more local voxels, if anything *strengthened* the benefit of functional searchlight.

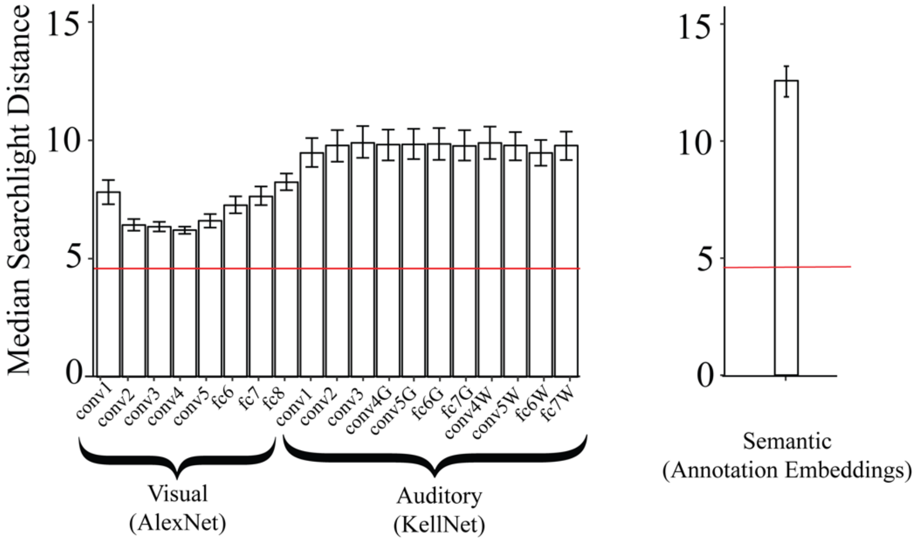

Fig S4. Anatomical distance between voxels within searchlights.
The median Euclidean distance (in voxel units) of the anatomical coordinates of voxels from within the top 1% of functional searchlights in the movie content experiment. Error bars denote 95% confidence intervals across subjects and the red line denotes the median Euclidean distance of voxels from within anatomical searchlights (constant determined by searchlight radius). An appropriate ceiling for these values is the median Euclidean distance in a searchlight consisting of randomly chosen brain voxels (approximately 28 voxels).

Additional manuscript changes:

> "Our analyses of the location of voxels from top-performing searchlights across individual subjects (Fig S3) and the median Euclidean distance between these voxels (Fig S4) indicate that the representations captured by functional searchlight were distributed and consistent across subjects. The fact that the advantage of functional searchlight over anatomical searchlight in visual and auditory analyses increased for a larger searchlight size (Fig S6) further suggests that the voxels in functional searchlight were broadly distributed rather than locally concentrated in larger regions." p. 8

> "Although the voxels from the top-performing searchlights in the semantic analysis were highly distributed across the cortex in every subject (Fig S3), the

spatial distribution of these voxels was not as consistent across subjects as the visual and auditory analyses." p. 9

3) While the paper is mainly very clearly written, I didn't understand what information was contained in the "S" matrix. The authors state (p. 13) that S is a k-by-t matrix "representing the shared response". It is clear that the SRM is encoding each individual subject's time-series data as a weighted combination of a common set of features at each time-point, but I don't know what the "shared-response" features are or how they were computed. Also, I think there is a typo in this paragraph, which describes W as a k-by-v matrix and S as a k-by-t matrix. I think one of those needs to be inverted in order to multiply these (like, maybe V is v-by-k).

We apologize for the confusing descriptions of the SRM equations and have rewritten them to be clearer.

Manuscript change:

"We model fMRI responses X (v voxels by t times data matrix) for subject i as $X_i = W_i S + E_i$. W_i (v by k features weight matrix) is subject-specific orthonormal bases representing individual functional topographies. S (k by t shared response matrix) is latent features that capture shared variance across all subjects. E_i (v by t error matrix) is the subject response not captured by the shared response to the common stimulus. SRM learns n (subjects) W_i orthogonal weight matrices and one S shared response matrix such that the quantity $\sum_{i=1}^{n} ||X_i - W_i S||_F$ is minimized." p. 14-15

4) It would be useful to see some discussion of how this approach relates to other approaches that likewise aim to find widely-distributed representational structure, such as plain old whole-brain regularized regression (as explored, for instance, by Irina Rish), the "generative modeling" approach pursued by Tom Mitchell, Jack Gallant etc, and the "structured sparsity" approaches (SOS LASSO, network RSA) developed by our group. I realize extensive discussion is out of bounds for a short paper, but a paragraph briefly sketching how the current approach relates to these would be v helpful.

We thank the reviewer for this helpful suggestion to build bridges to other related methods. We have added a discussion paragraph as requested.

Manuscript change:

"There are several other powerful methods for multivariate fMRI that can pick up on distributed representations. For example, regularized whole-brain methods [20] use all voxels in the brain as features for a regression, but put a penalty on the matrix norm of the regression's feature weights. In other words, all brain voxels are considered, but the space of solutions of how voxels are weighted is restricted to those in which the vast majority of weights are either very small or 0 and only a sparse set of voxels are the main drivers of the regression. Sparse-overlapping Sets (SOS) Lasso extends this idea using structured sparsity. In SOS Lasso, neighboring voxels within a specific radius are grouped into sets and, instead of having weights on individual voxels that contribute to the sparsity penalty, weights are put on these voxel sets for the sparsity penalty. PrAGMATiC [22] is a similar approach that instead uses probabilistic groupings.

These powerful methods generally utilize free parameters that need to be selected through cross-validation or other computationally intensive routines. For example, regularized whole-brain methods [20] require choosing a regularization function (L1 or L2) and fitting of a penalty parameter, both of which have to be optimized through cross-validation grid search. SOS Lasso also has choices for penalty terms and free parameters [21]. PrAGMATiC [22] requires the fitting of

extra parameters through a Markov Chain Monte Carlo (MCMC) framework. Functional searchlight with SRM does not involve any free parameters, nor assumptions about the sparsity of representations in the brain. That said, it does assume that the stimulus used to construct the functional space will rearrange the voxels in a way that is relevant for subsequent analyses. It is possible that in some domains, such as for complex cognitive control tasks, rearranging voxels based on passive movie viewing will not improve analyses of those tasks. Future work should investigate the boundary conditions of tasks that can benefit from functional searchlight.” p. 12-13
