# Supplementary material for: Searching through functional space reveals distributed visual, auditory, and semantic coding in the human brain
Source: PLoS Comput Biol. 2020 Dec 3;16(12):e1008457. doi: 10.1371/journal.pcbi.1008457 (PMC7738169; doi:10.1371/journal.pcbi.1008457)
Supplement: Attachment — Submitted filename: Kumar_PCB_Revision2_Letter_Final.pdf [file pcbi.1008457.s011.pdf]

Dear Drs. Jbabdi and Marinazzo,

We are pleased that our manuscript "Searching through functional space reveals distributed visual, auditory, and semantic coding in the human brain" was deemed likely to be accepted pending final revisions. Below we provide point-by-point responses to the remaining comments from one reviewer and highlight where in the manuscript changes were made. We look forward to hearing from you about next steps.

Thanks,

Nick Turk-Browne (on behalf of all authors)

--

Reviewer #1: Thanks for addressing my concerns and taking pains to run control experiments and clarify some misconceptions such as signal filtering.. I apologize if my comments came out strong which wasn't my intention but to express my concerns with the interpretation and claims of novelty of some aspects of the paper.

> We want to thank the reviewer for their prior and current feedback, which greatly strengthened our work.

Few minor questions: how do the functional searchlights look across the two studies (Forrest and Sherlock). Do they reveal similar distributed patterns across studies? This is related to my original concern of whether these are revealing some general distributed networks comparable across experiments.

> This is an interesting question worth addressing in the manuscript. The movies differ in terms of style, characters, dynamics, emotions, etc., so we did not expect that they would necessarily drive brain activity in a way that the features discovered by SRM would be identical or highly similar. For example, an SRM feature (or set of features) could correspond to individual actors, speaker accents, or locations present in the Sherlock episode but not in Forrest Gump. If so, then voxels that organize along such dimensions in functional space would not be consistent across movies. Nonetheless, we did expect that some features might be present in both movies and thus that there would be some similarity between their functional spaces in the aggregate. For example, such features could respond more generically to faces, language, or buildings.
>
> To test this possibility, we conducted a new analysis in which we compared the pairwise distances between voxels in the functional spaces derived from the Sherlock and StudyForrest datasets. For each dataset, the vector mappings of voxels into the features of the shared response were used to calculate the Euclidean distance between all pairs of voxels. The similarity of functional spaces between datasets was then calculated as the correlation of these distances across voxel pairs. Because there is an astronomical number of such pairs, we used a Monte Carlo approach of calculating the correlation of distances in a random sample of voxel pairs and repeating several times to estimate the overall correlation. We tested statistical significance by comparing this value to a null distribution of correlations generated by randomization. The pairwise distances had a modest but highly significant positive correlation across datasets, suggesting that the functional spaces learned from the movies were at least somewhat similar.
>
> Manuscript change:

"Additionally, we tested whether the functional spaces generated from the Sherlock movie data and the StudyForrest movie data were similar. We found that the pairwise distances between voxels in these spaces had a modest but highly significant positive correlation (Fig S8). This suggests that SRM can capture a general organization of voxels common across audiovisual movie viewing conditions, but also that individual movie content may partly govern the learned functional spaces." p. 11-12

Added Figure S8:

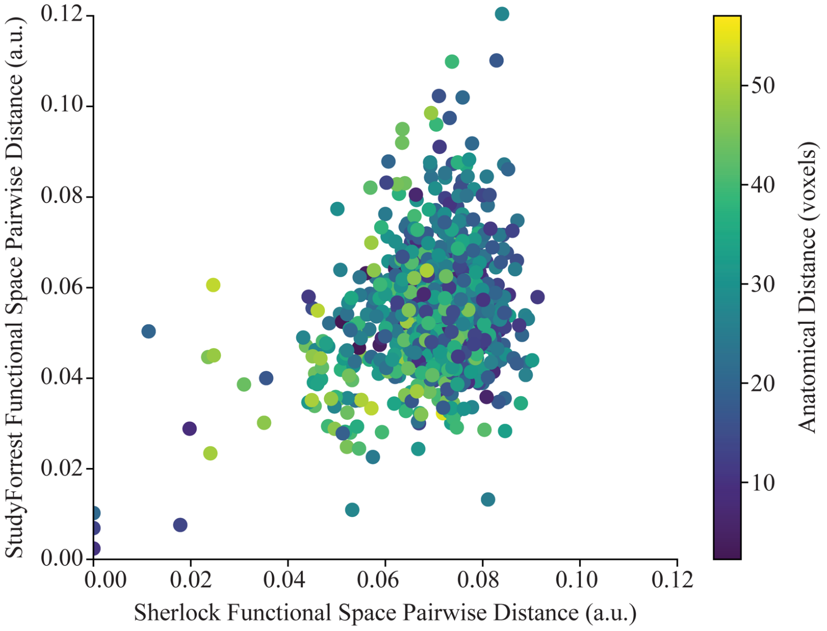

Fig S8. Comparing SRM functional spaces from different datasets.
To determine the similarity of the functional spaces constructed by SRM for the Sherlock and StudyForrest datasets, we extracted the Euclidean distances between all voxel pairs in functional space and then correlated these distances between datasets. The overall correlation was estimated by randomly sampling the same 10,000 voxel pairs from the two datasets, calculating the correlation of distances in the sample, repeating 1,000 times with new random samples, and then averaging across iterations. There was a positive correlation overall (r = 0.198). To test statistical significance, we performed a non-parametric randomization test by repeating this process 1,000 times while randomly permuting the voxel distances of the Sherlock dataset each time to populate a null distribution. The true correlation of the datasets was highly significant (p < 0.001). One of the samples used to estimate the overall correlation is visualized in the plot, with each dot depicting one voxel pair and the axes corresponding to their Euclidean distance in the two functional spaces. Color coding by anatomical distance of each voxel pair indicates how dramatically SRM reshaped the brain based on function (i.e., voxels that are anatomically distant can be functionally close, and vice versa). a.u. indicates arbitrary units.

- Results section still doesn't contain numbers but just qualification of them. You can specify the average increase in correlations and accuracies in the text to give the reader an idea of effect sizes. There is no way to parse if from the text now. I also suggest using actual correlation and classification accuracy values as the main figure (S5 now) rather than the percent changes which will be a more direct report of results.

We appreciate the interest of the reviewer in presenting the data more directly. As suggested, we added a new figure (Figure 3) based on the old Figure S5. Because this figure reports all neural network layers and the main percent change figure depicts only

representative layers, we have also added to the new figure the percent change for all layers from the old Figure S2. Additionally, we report the requested numerical results in the main text.

Added Figure 3:

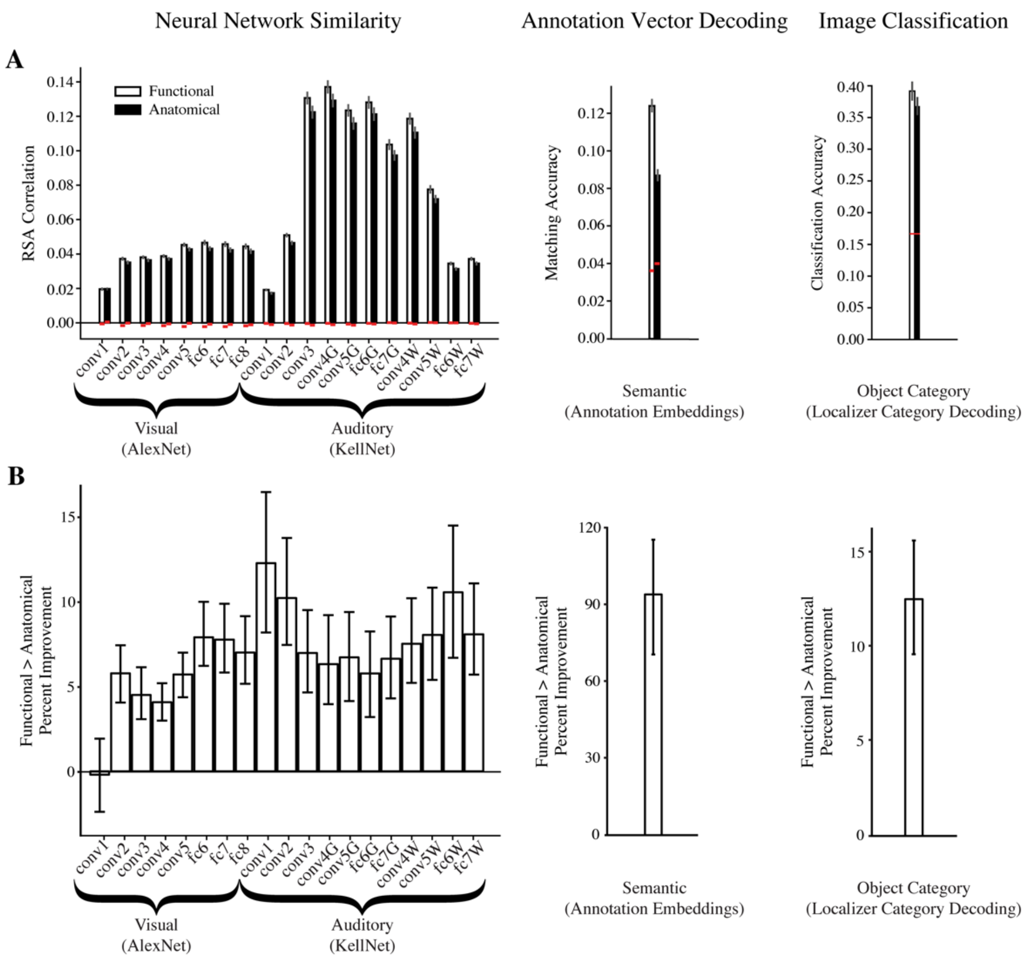

Fig 3. Raw accuracy and percent improvement for all analyses.
(A) Average performance in the top 1% of functional and anatomical searchlights for neural network similarity, annotation vector decoding, and localizer category decoding. White bar is the functional searchlight performance, black bar is the anatomical searchlight performance. Error bars represent standard error across subjects. Chance (red lines) was computed in the neural network similarity and annotation vector decoding analysis as the mean of a null distribution estimated non-parametrically by rolling data in time, and in image classification as the theoretical chance level (1/6 categories). (B) Percent improvement of functional over anatomical searchlight in the top 1% of searchlights for neural network similarity (all layers), annotation vector decoding, and localizer category decoding. To calculate percent improvement, we first subtracted the chance level from the performance of each searchlight type. Error bars represent 95% CIs.

Additional manuscript changes:

> "The average increase in RSA collapsing across layers was 5.433% for AlexNet (95% CI = [3.709, 7.224], bootstrap $p < 0.0001$) and 8.273% for KellNet ([5.423, 11.355], $p < 0.0001$)." p. 8

"The average increase in annotation vector decoding was 94.155% (95% CI = [70.819, 116.024], bootstrap $p < 0.0001$)." p. 9

"The average increase in image classification was 12.576% (95% CI = [9.615, 15.917], bootstrap $p < 0.0001$)." p. 11

- Clarify that the functional searchlights are derived from the movie and applied to the localizer experiment in text.

We agree that this needed to be clearer and have modified the text.

Manuscript change:

"We ran functional and anatomical searchlight analyses to decode localizer image category from voxel activity patterns (six categories: bodies, faces, houses, small objects, landscapes, scrambled images). As before, the functional space was learned from SRM of movie data (Forrest Gump). However, the resulting functional searchlights were now applied to a separate localizer task with blocks of the different categories. We found that the functional searchlight reliably outperformed anatomical searchlight (Fig 2C, Table S1)." p. 11
